# Quercetin Protects against Okadaic Acid-Induced Injury via MAPK and PI3K/Akt/GSK3β Signaling Pathways in HT22 Hippocampal Neurons

**DOI:** 10.1371/journal.pone.0152371

**Published:** 2016-04-06

**Authors:** Wei Jiang, Tao Luo, Sheng Li, Yue Zhou, Xiu-Yin Shen, Feng He, Jie Xu, Hua-Qiao Wang

**Affiliations:** 1 Department of Anatomy and Neurobiology, Zhongshan School of Medicine, Sun Yat-sen University, Guangzhou, Guangdong, 510080, China; 2 Department of Anatomy and Histoembryology, Zhaoqing Medical College, Zhaoqing, Guangdong, 526020, China; 3 School of Pharmaceutical Science, Sun Yat-sen University, Guangzhou, Guangdong, 510006, China; Massachusetts General Hospital/Harvard Medical School, UNITED STATES

## Abstract

Increasing evidence shows that oxidative stress and the hyperphosphorylation of tau protein play essential roles in the progression of Alzheimer’s disease (AD). Quercetin is a major flavonoid that has anti-oxidant, anti-cancer and anti-inflammatory properties. We investigated the neuroprotective effects of quercetin to HT22 cells (a cell line from mouse hippocampal neurons). We found that Okadaic acid (OA) induced the hyperphosphorylation of tau protein at Ser199, Ser396, Thr205, and Thr231 and produced oxidative stress to the HT22 cells. The oxidative stress suppressed the cell viability and decreased the levels of lactate dehydrogenase (LDH), superoxide dismutase (SOD), mitochondria membrane potential (MMP) and Glutathione peroxidase (GSH-Px). It up-regulated malondialdehyde (MDA) production and intracellular reactive oxygen species (ROS). In addition, phosphoinositide 3 kinase/protein kinase B/Glycogen synthase kinase3β (PI3K/Akt/GSK3β) and mitogen activated protein kinase (MAPK) were also involved in this process. We found that pre-treatment with quercetin can inhibited OA-induced the hyperphosphorylation of tau protein and oxidative stress. Moreover, pre-treatment with quercetin not only inhibited OA-induced apoptosis via the reduction of Bax, and up-regulation of cleaved caspase 3, but also via the inhibition of PI3K/Akt/GSK3β, MAPKs and activation of NF-κB p65. Our findings suggest the therapeutic potential of quercetin to treat AD.

## Introduction

Alzheimer’s disease (AD) is a progressive neurodegenerative disease which is characterized by short-term memory loss, language and speech difficulties and disorientation. The pathological changes of AD are the deposition of β-amyloid, neurofibrillary tangles (hyperphosphorylation of tau protein) and oxidative damage [1].

Clinical evidence indicates the number of neurofibrillary tangles was positively correlated with the severity of dementia in AD patients [2]. Studies have shown that oxidative stress promotes AD progression [1, 3]. OA, isolated from black sponge Holichondria okadai, is a potent selective inhibitor to serine/threonine protein phosphatases 1 and 2A [4] and can induce oxidative stress and hyperphosphorylation of tau, thus result in AD damage [3, 5].

**Fig 1:**
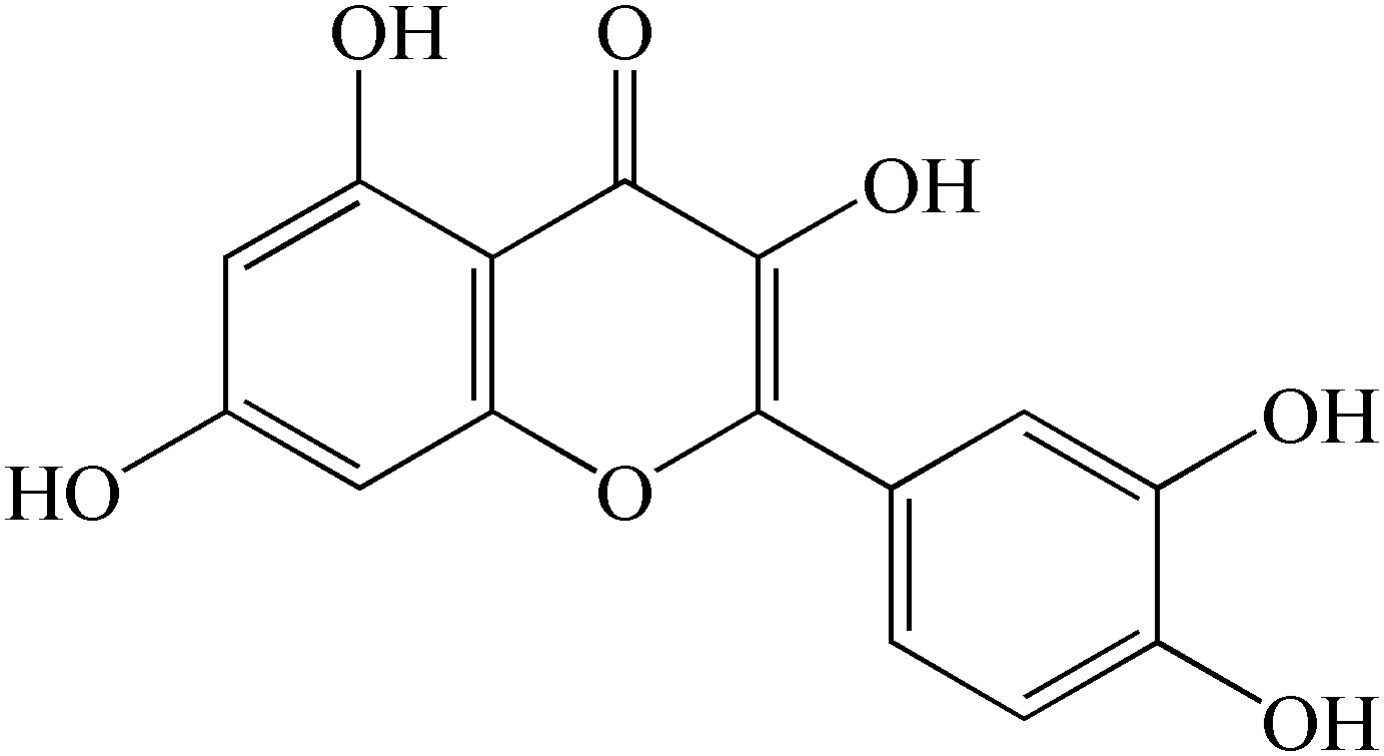
Chemical structure of quercetin.

Some antioxidants has been shown to protect neurons from oxidative damage [6, 7]. Quercetin is an emerging compound from flavonoid that can scavenge free radicals (Fig 1). Pharmacodynamics revealed that quercetin improved cognitive function in the APP/PS1 transgenic mouse model of AD [8] and protected PC12 cells from Aβ-induced toxicity [9]. Islam and colleagues showed that quercetin has the therapeutic potential to treat AD by silico QSAR analysis [10].

However, the neuro-protective effects of quecetin on tau phosphorylation and the underlying mechanisms remain unclear. To determine the therapeutic potential of quercetin to AD, we investigated the anti- hyperphosphorylation of tau protien, anti-oxidative stress and anti-apoptotic effects of quercetin on HT22 cell line after exposure to OA. Furthermore, the underlying signaling pathways, such as MAPK and PI3K/Akt/GSK3β, for these effects were studied.

## Materials and Methods

### Materials

Quercetin (C_15_H_10_O_7_, FW: 302.24, Purity > 98%) was obtained from Aladdin Industrial Corporation (Shanghai, China). Okadaic acid (OA), Rhodamine123 (Rh123), Dimethylsufoxide (DMSO), 2′,7′- Dichlorofluorescin diacetate (DCFH-DA), and LiCl were purchased from Sigma-Aldrich (Saint Louis, MO, USA). Cell Counting Kit-8 (CCK-8) was purchased from Dojindo (Kumamoto, Japan). Fetal bovine serum, 100 kU/L penicillin and 100 mg/L streptomycin were bought from Gibco-BRL Invitrogen (San Diego, USA). BCA protein Assay Kit was purchased from Beyotime institute of Bioengineering (Jiangsu, China). The reagent kits for the measurement of LDH, MDA, SOD, and GSH-Px were purchased from Nanjing Jiancheng Bioengineering Institute (Nanjing, China). Protease inhibitor cocktail and phosphatase inhibitor cocktail were purchased from Merk-Millipore (MA, USA). Anti-cleaved caspase-3 and anti-β-actin were obtained from Cell Signaling Technology (Boston, USA). Anti-Bax was purchased from Abcam (Cambridge, UK). Horseradish peroxidase-conjugated anti-rabbit antibody was shipped from Bioworld Technology CO., Ltd (Minneapolis, USA). All the phosphorylated antibodies used in this study were summarized in Table 1.

**Table 1:** Phosphorylated antibodies used in this study.

Antibody	Specificity	Type	Dilution	Source
Tau-1	Dephosphorylated tau	mAb	1: 10000	Millipore
pS199	Phosphorylated tau at Ser199	pAb	1: 1000	Invitrogen
pT205	Phosphorylated tau at Thr205	pAb	1: 1000	Invitrogen
pS396	Phosphorylated tau at Ser396	mAb	1: 2000	Abcam
pT231	Phosphorylated tau at Thr231	mAb	1: 1000	Abcam
Tau-5	Total-tau protein	mAb	1: 1000	Abcam
GSK3β	Total GSK-3β	mAb	1: 2000	Abcam
GSk 3β (pY216)	Phosphorylated GSK-3β at Tyr216	pAb	1: 1000	Abcam
GSk 3β (pSer9)	Phosphorylated GSK-3β at Ser9	pAb	1: 500	Abcam
AKT	Total AKT	mAb	1: 1000	CST
p-AKT	Phosphorylated AKT at Ser473	mAb	1: 2000	CST
ERK1/2	Total ERK	mAb	1: 1000	CST
p-ERK1/2	Phosphorylated ERK at Thr202/Tyr204	mAb	1: 1000	CST
JNK	Total JNK	mAb	1: 1000	CST
p-JNK	Phosphorylated JNK at Thr183/Tyr185	mAb	1: 1000	CST
p38	Total P38	mAb	1: 1000	CST
p-p38	Phosphorylated p38 at Thr180/Tyr182	mAb	1: 1000	CST
NF-κB p65	Total p65	mAb	1: 1000	CST
NF-κB p-p65	Phosphorylated p65 at Ser536	mAb	1: 1000	CST

pAb: polyclonal antibody; mAb: monoclonal antibody; CST: Cell signaling technology

### Cell Culture

The HT22 cells were a generous gift from Dr. Jun Liu (The Memorial Hospital of Sun Yat-Sen University, Guangzhou, China) [11]. The cell line is a subclone of HT4, which was derived from the mouse hippocampus [12]. The cells were cultured in DMEM supplemented with 10% (v/v) FBS, 100 kU/L penicillin, and 100 mg/L streptomycin at 37°C in an atmosphere containing 5% CO_2_ and 95% air.

### Cell Treatment

OA was dissolved in DMSO to obtain 200μmol/L stock solution, and was diluted into sequential concentrations with serum-free media before using. Quercetin was dissolved and diluted with DMSO to obtain 100mM stock solution. HT22 cells were treated with various concentrations (20-160nmol/L) of OA for 12h to optimize the appropriate concentration of drug.

HT22 cells were pre-treated with quercetin (5, 10μmol/L) for 12 h, then 80nmol/L OA was added and incubated for another 12h in fresh media. To investigate the neuroprotective mechanism of quercetin, we had several experimental groups: 5μmol/L quercetin, 10μmol/L LY294002 (PI3K inhibitor), 10mmol/L LiCl (GSK3β inhibitor), quercetin + LY294002 and quercetin + LiCl. HT22 cells were incubated with these treatments for 12h in before incubation with OA (80nmol/L) for 12 h., In addition, HT22 cells were treated with 10μmol/L LY294002 alone for 24h after growing 12h.

### Analysis of Cell Viability (CCK-8 assay)

Cell viability was assessed by the CCK-8 Cell Counting kit. In brief, HT22 cells were seeded in 96-well plates with a density of 5000 cells/well. The cells were pre-treated with sequential concentrations of quercetin (0, 2.5, 5.0, 10.0, and 20.0μmol/L) for 12 h, and 80nmol/L OA was added to each group for 12h incubation. We also measured the cytotoxicity of quercetin at various concentrations (0, 5, 10, 20, 50, 100μmol/L). Finally, the absorption at 450nm was measured by microplate reader (TECAN, Austria).

### Determination of LDH Release, Lipid Peroxidation Products and Antioxidant Enzyme Activity

OA (80nmol/L) and quercetin (5μmol/L, 10μmol/L) were added as described above at 70–80% confluence. Control group was treated with 0.5% DMSO. Cells were lysed on ice for 30min and centrifuged at 12, 000g for 10min at 4°C. The supernatants were separated. Briefly, MDA was evaluated using the thiobarbituric-acid-reactive substancemethod [5]. The cellular antioxidant enzyme (Cu-Zn, SOD and GSH-Px) activity was measured using the commercially available colorimetric assay kit (Nanjing Jiancheng Bioengineering Institute). Some cells were centrifuged and LDH activity was measured using the LDH assay kit (Nanjing Jiancheng Bioengineering Institute).

### Detection of Reactive Oxygen Species (ROS)

HT22 cells were plated on 24-well plates (cell density, 3×10^4^/well). Cells were treated as previous described. DCFH-DA in serum-free DMEM was added at a final concentration of 10μM for 30 min at 37°C. The intracellular oxidant productions were measured. Mean intensity of fluorescence for randomly selected 3 fields were measured and analyzed by Image J 1.41o software (NIH, USA).

### Measurement of Mitochondrial Membrane Potential (MMP)

The mitochondrial membrane potential was measured by using rhodamine123 fluorescent dye [6]. HT22 cells were incubated with rhodamine123 (2μmol/L) for 30min at 37°C for all the above groups. After incubation, cells were washed with PBS for three times. Fluorescent images were taken (Leica, Germany). All data were analyzed using Image J 1.41. software (NIH, USA).

### Western Immunoblotting

Protein levels were measured using western blotting as previously described [7]. Briefly, cells were lysed for 30 minute in ice-cold RIPA solution (1× PBS, 1% NP40, 0.1% SDS, 5mmol/L EDTA, 0.5% Sodim Deoxycholate, 1% Protease inhibitor and 1% phosphatase inhibitor). The homogenate was centrifuged at 12000×g for 10 min at 4°C. The supernatant was collected and protein concentrations were measured using BCA Protein Assay Kit. After adding loading buffer, samples with equal amount of protein were electrophoresed on 10%, 12% or 15% SDS-polyacrylamide gel. Proteins (about 30μg) were transferred to polyvinylidene difluoride membranes. The membranes were blocked with 5% bovine albumin in TBST (TBS and 0.1% Tween 20) for 1 h at room temperature. The blots were incubated overnight with anti-β-actin (1:1000), anti-cleaved caspase-3 (1:1000), anti-Bax (1:1000). Then after washing three times with TBST, the membranes were incubated with HRP-conjugated antibody (1:10000) for 1h at room temperature and were washed for three times with PBS. Bands were visualized using ECL detection reagents. The gray values of sample bands were quantified using Image J 1.41. and normalized by β-actin.

### Statistical Analysis

Data were analyzed by SPSS 16.0 statistical software package using one-way analysis of variance (ANOVA) followed by LSD’s post hoc test. All data were presented as mean ± S.E.M The criterion of significance was *P* < 0.05.

## Results

### Effects of OA on Phosphorylation of Tau Protein via GSK3β and p38-MAPK

**Fig 2:**
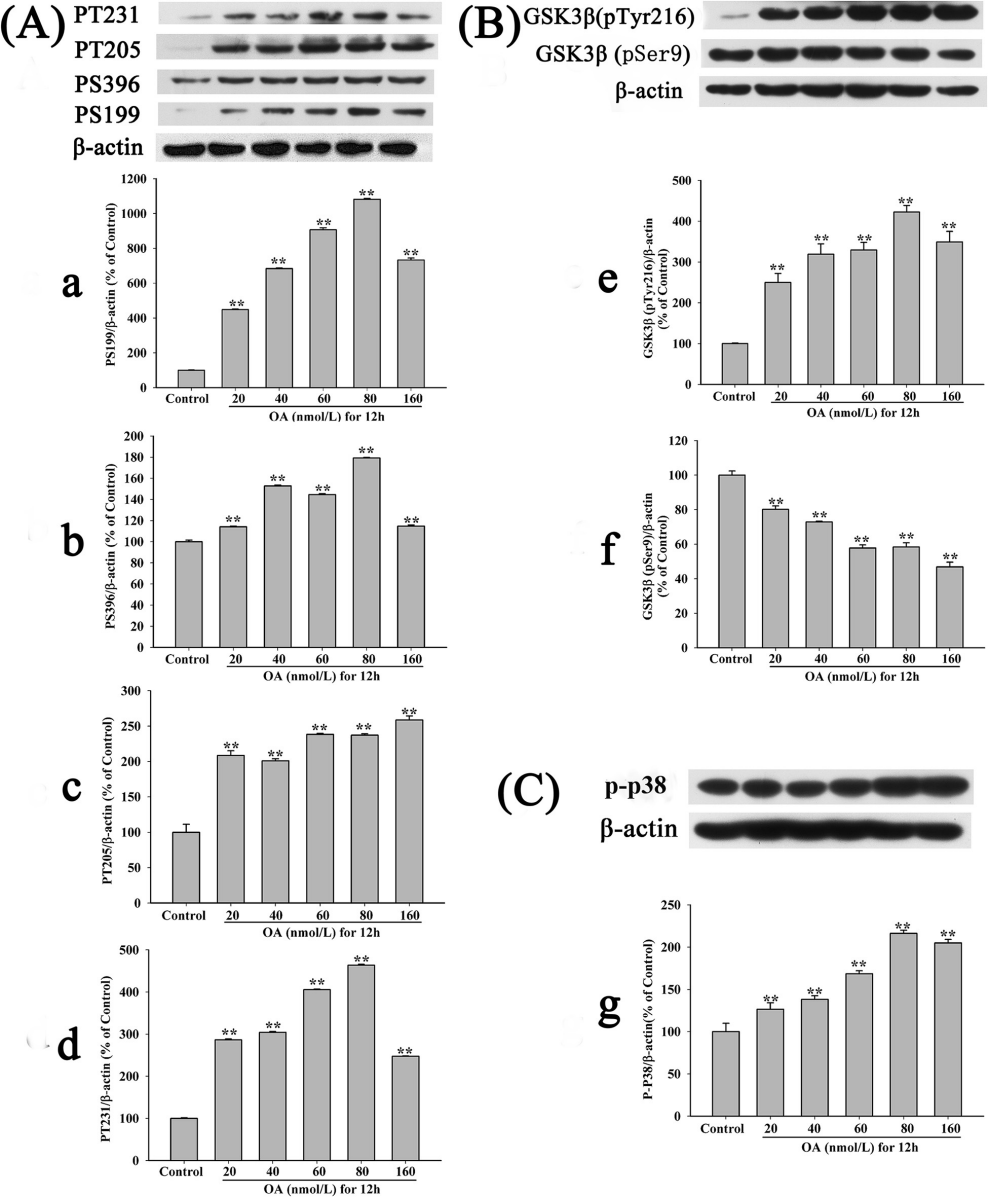
Effects of OA on the phosphorylation of tau protein. Cell lysates were treated with various concentrations of OA (20-160nmol/L) for 12h. Protein levels of different phosphorylated tau protein, GSK3β and p38-MAPK were analyzed by western blotting, which were phosphorylated tau (**A**), phosphorylated GSK3β to pTyr216 and pSer9 (**B**) and phosphorylated p38-MAPK (**C**). Quantitative analysis of the blots showed in panel **a-g**. Data normalized by β-actin were analyzed by ANOVA with LSD’s post-hoc test (**P < 0.01 *vs*. control group).

In this study, we quantified the levels of tau phosphorylation at different sites by western blotting. Cells were incubated with various concentrations of OA (0, 20, 40, 60, 80, 160nmol/L) for 12h. As shown in Fig 2, the phosphorylation of tau, p38-MAPK and GSK3β had a significant increase in a dose-dependent manner after treatment with OA (20–80 nmol/L). The phosphorylation of tau protein at Thr231, Thr205, Ser199 and Ser396 was peaked when treated with OA at 80nmol/L. p38 MAPK and GSK3β also were maximal activated at this concentration. The phosphorylation of tau at different sites and activation of p38-MAPK were increased by 363.57% (Fig 2A and 2a),137.20%, (Fig 2A and 2b), 982.40% (Fig 2A and 2c), 79.26% (Fig 2A and 2d) and 116.27% (Fig 2C and 2g) respectively compared to DMSO-treated control; The phosphorylation of active site at Tyr216 (Fig 2B and 2e) increased by 322.38% and the inhibitory site at Ser9 (Fig 2B and 2f) decreased by 41.65% in GSK3β. However, the phosphorylation of tau and activities of p38-MAPK and GSK3β were reduced by OA at 160nmol/L. Therefore, 80nmol/L of OA was chosen as optimal concentration for the following experiments.

### Effects of Quercetin on OA-Induced Neurotoxicity

**Fig 3:**
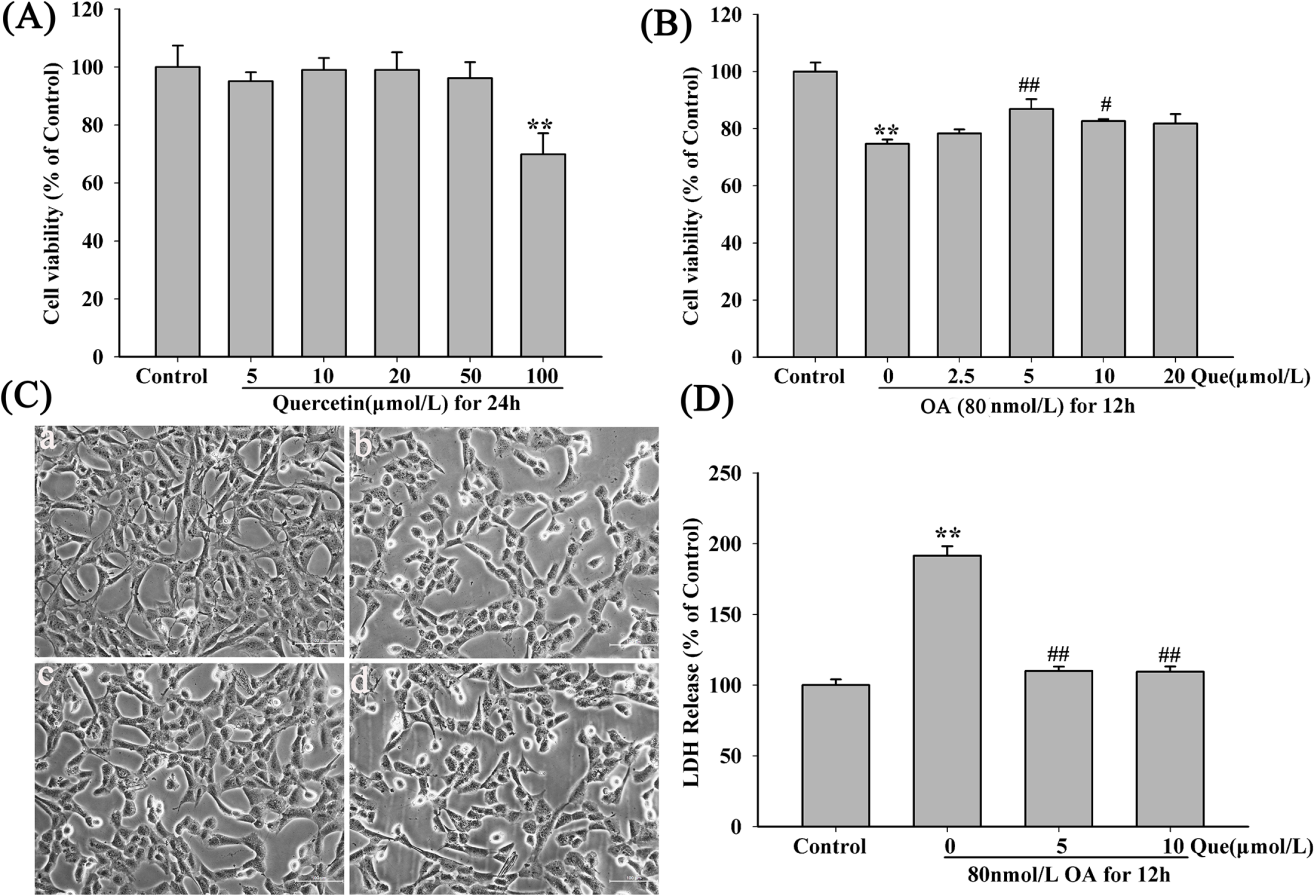
Effects of quercetin on OA-induced neurotoxicity. (**A**) Final concentration of DMSO in the mediumwas 0.5% (the control was also included). The quercetin did not affect the cell viability of HT22 cells after incubation for 24h with different concentrations(<100μmol/L); (**B**) Cells were pre-treated with different concentrations of quercetin for 12h before incubation of 80nmol/L OA for 12 h. The cell viability was measured by CCK-8 assay; (**C**) Morphology of HT22 cells was injured by OA but was reversed by quercetin (200x, 5, 10μM); (**a**) Control group; (**b**) 80nmol/L OA; (**c**) 5μmol/L quercetin + 80nmol/L OA; (**d**) 10μmol/L quercetin + 80nmol/L OA; Scale bar = 100μm. (**D**) Pre-treated with quercetin (5.0, 10.0μmol/L) for 12h decreased LDH release which was increased by OA at 80nmol/L. (n = 5, **P < 0.01 *vs*. control, ^#^*P* < 0.05, ^##^*P* < 0.01 *vs*. OA-control)

No reduction of cell viability was observed when treated with quercetin (Fig 3A), indicating no detectable cytotoxicity (concentration< 100μmol/L) of the drug. However, pre-treated with various concentrations of quercetin (2.5, 5.0, 10.0, 20.0μmol/L) for 12h increased the cell viability to 78.36 ± 2.48%, 86.88 ± 6.02%, 82.62 ± 1.28% and 81.79 ± 5.78%, compared to OA group (80nmol/L, 74.72 ± 2.56%) (Fig 3B), and cell morphology was apparently improved in groups pre-treated with quercetin (Fig 3C). We also found that the secretion of LDH was significantly decreased after pre-treatment with quercetin (5,10μmol/L) (Fig 3D). Therefore, we used 5μmol/L and 10μmol/L of quercetin in the following experiments.

### Quercetin Blocked OA’s Effects on MDA, Cu-Zn SOD and GSH-Px

The level of MDA markedly increased by 109.62% (P< 0.01), when incubated with 80nmol/L OA for 12h compared to control group (Table 2). However, this effect was reversed by pre-treatment of quercetin (5,10μmol/L) (Table 2). The level of GSH and activity of SOD were significantly decreased 16.98% and 24.93% respectively compared to control group in OA group (Table 2). Interestingly, the decreases were attenuated by pre-treatment of quercetin (Table 2).

**Table 2:** Effects of different concentrations of quercetin on change of MDA, SOD and GSH-Px induced by OA in HT22 cells.

Groups	MDA(nmol/mg Protein)	SOD(U/mg Protein)	GSH-Px(U/mg Protein)
**Control**	0.20 ± 0.07	10.28 ± 0.06	577.83 ± 17.18
**OA**	0.42 ± 0.09**	7.72 ± 0.05**	479.59 ± 5.92**
**Quercetin (5μmol/L)+OA**	0.26 ± 0.09^##^	10.24 ± 0.08^##^	595.19 ± 16.77^##^
**Quercetin (10μmol/L)+OA**	0.25 ± 0.06^##^	9.25 ± 0.07^##^	557.52 ± 18.71^##^

(**P < 0.01 *vs*. control group, ^##^*P* < 0.01 *vs*. OA-control group)

### Effects of Quercetin on Intracellular Level of ROS

**Fig 4:**
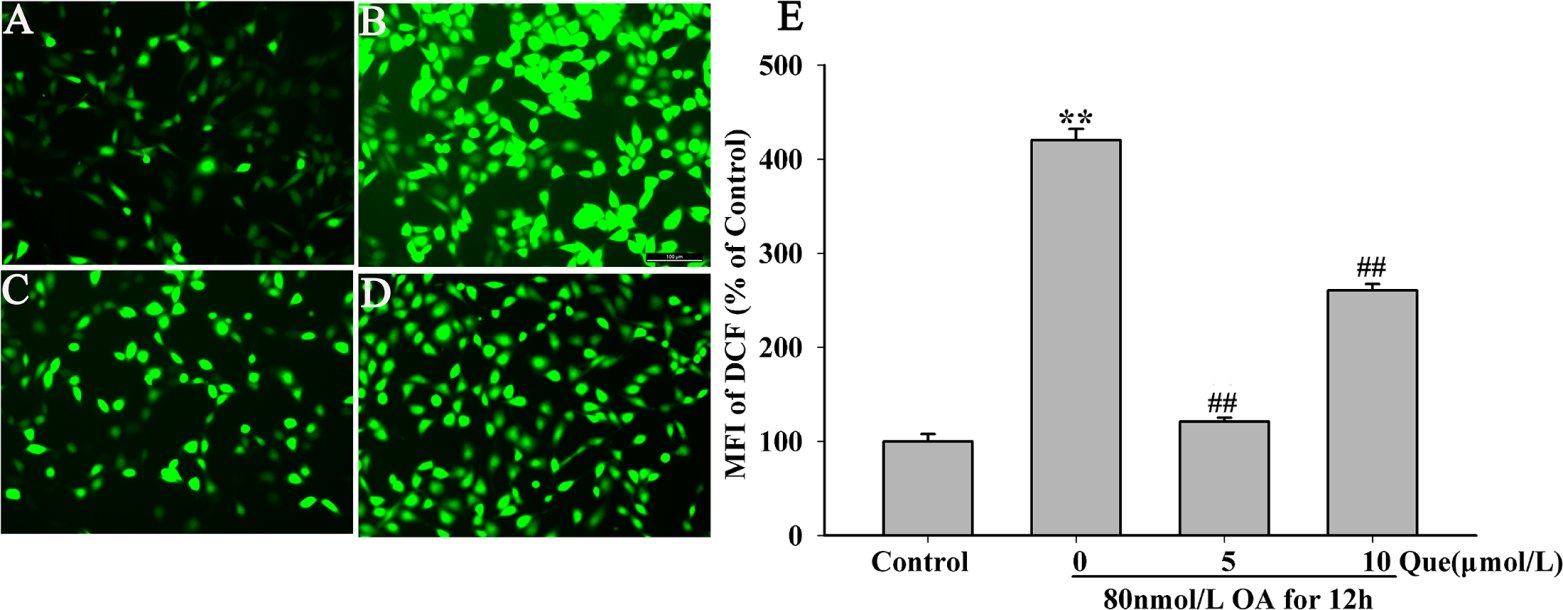
Effects of different concentrations of quercetin on OA induced ROS production in HT22 cells (200×). Quercetin was added 12 h prior to OA induction and cells were incubated for another 12 h with OA. The ROS was quantified as previously described with the mean intensity of fluorescence. (**A**) control group; (**B**) 80nmol/L OA; (**C**) 5μmol/L quercetin + 80nmol/L OA; (**D**) 10μmol/L quercetin + 80nmol/L OA and (E) quantitative analysis of mean intensity of fluorescence (**P < 0.01 *vs*. blank control, ^##^*P* < 0.01 *vs*. OA-control).

As described above, OA induction (80nmol/L) increased the intracellular leve of ROS compared to the control group (Fig 4B and 4E), and the quercetin (5,10μmol/L) markedly reduced the increase (Fig 4C–4E). These findings may indicate that quercetin protected OA induced cell injury to HT22 cells.

### Effects of Quercetin on Membrane Potential

**Fig 5:**
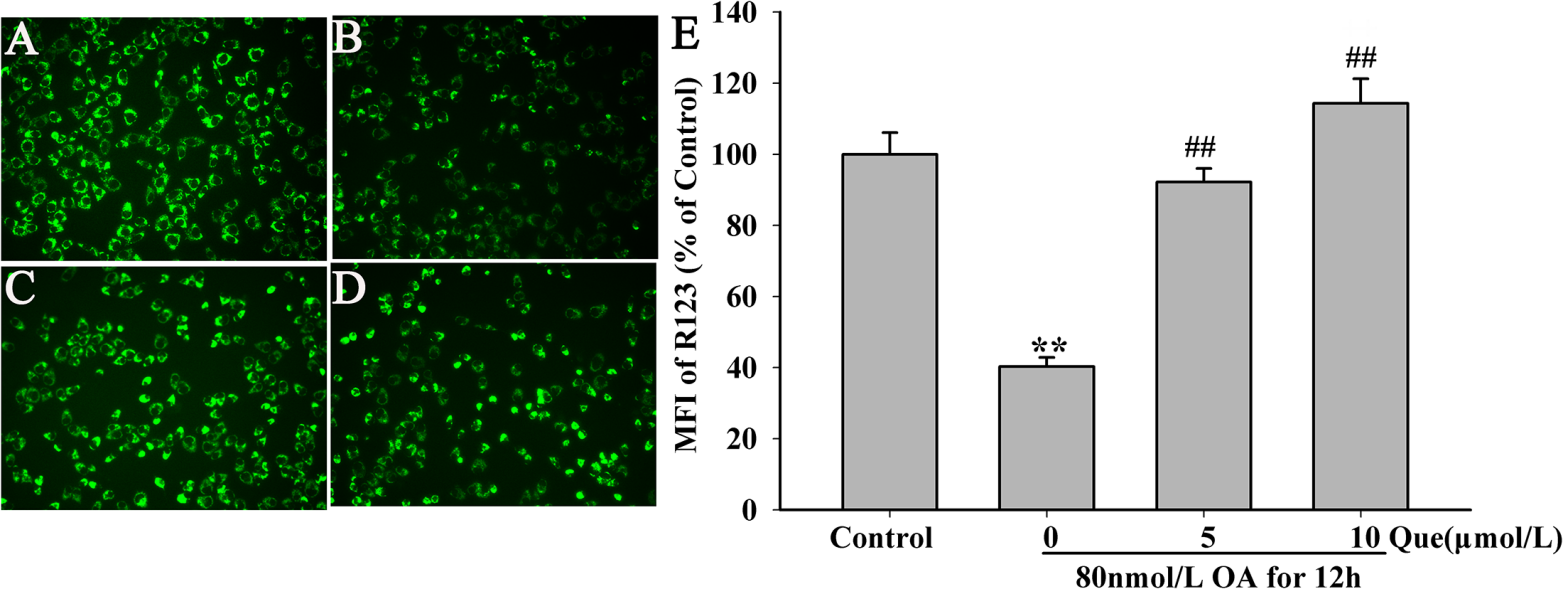
Quercetin reversed OA induced decrease of MMP in HT22 cells (200×). Quercetin was added 12h before OA incubation. The MMP was quantified as the mean intensity of fluorescence. (**A**) Control group; (**B**) 80nmol/L OA; (**C**) 5μmol/L quercetin + 80nmol/L OA; (**D**) 10μmol/L quercetin + 80nmol/L OA; (**E**) quantitative analysis of the mean fluorescent intensity (**P < 0.01 vs. control, ^##^*P* < 0.01 *vs*. OA-control).

The mitochondrial membrane potential (MMP) was evaluated using the fluorescent rhodamine dye, Rh123. As showed in Fig 5B and 5E, the MMP level was reduced to 59.70% of the control (*P* < 0.01) when OA (80nmol/L) was added and incubated for 12h. Quercetin (5, 10μmol/L) significantly reversed the OA-induced the decrease of MMP (Fig 5C–5E). These results (The raw data was showed in S1 Fig) indicate that quercetin may play an important role in protecting HT22 cells against OA-induced injury via the enhancement of MMP.

### Effects of Quercetin on OA-Induced Hyperphosphorylation of Tau Protein

**Fig 6:**
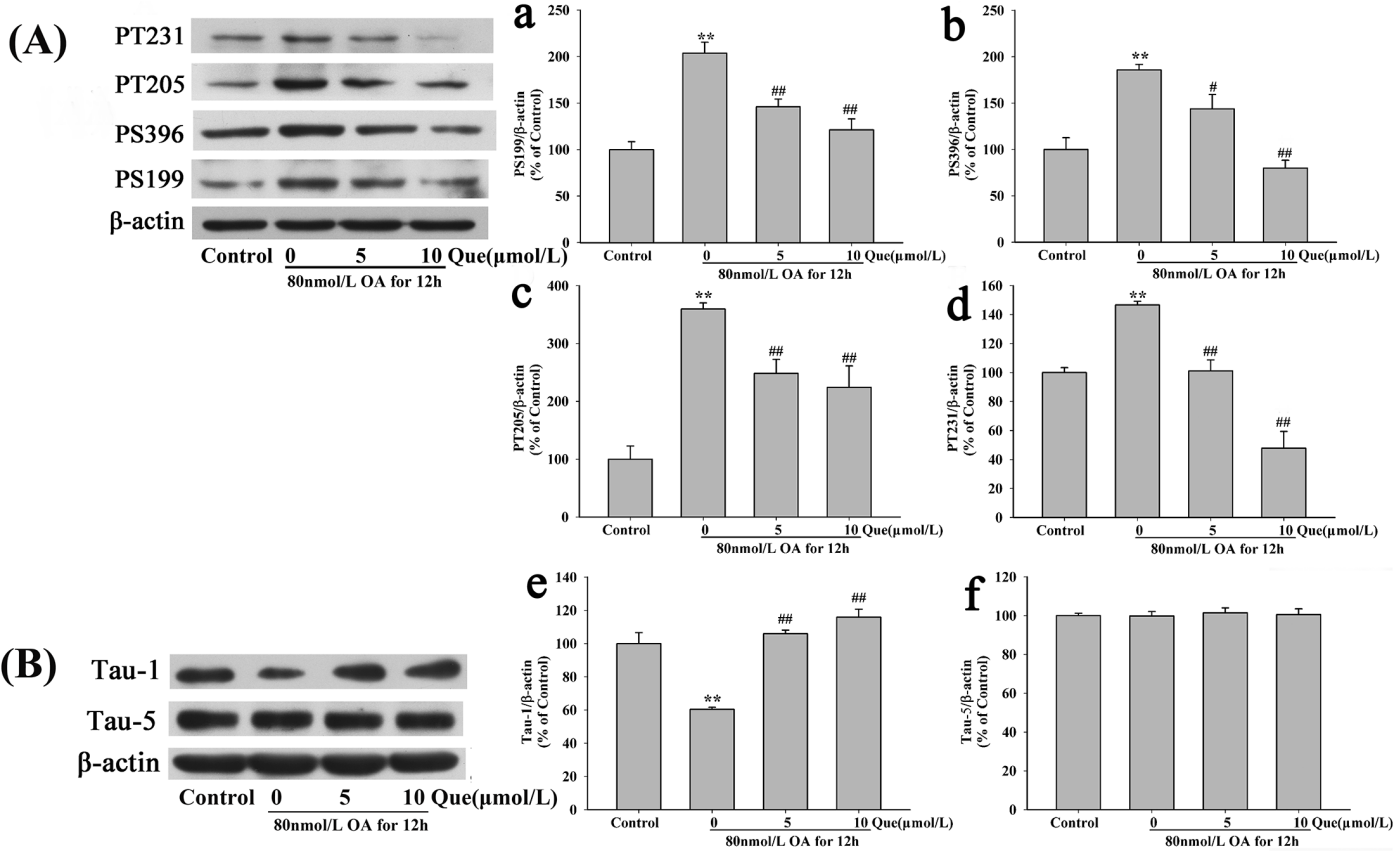
Effects of quercetin on OA induced hyperphosphorylation of tauprotein. Protein levels of phosphorylated tau proteins and total tau protein (Tau-5) were measured by western blotting (**A**, **B**). Statistical analysis for protein levels was shown in panel **a-f**. (**P < 0.01 *vs*. control, ^#^*P* < 0.05, ^##^*P* < 0.01 *vs*. OA-control).

Phosphorylated tau is a major neuropathological character of AD. In the current study, we investigated whether quercetin had any effects on OA-induced phosphorylation of tau protein. Compared to control group, treatment with OA (80nmol/L) for 12h obviously increased the levels of tau phosphorylation at pS396, pS199, pT205, and pT231 but decreased the level of phosphorylation at Tau-1. However, the treatment did not affect the level of total tau (Tau-5) (Fig 6B and 6f). Pre-treatment with quercetin (5,10μmol/L) for 12 h significantly suppressed OA-induced hyperphosphorylation of tau protein at pS396 (Fig 6A and 6a), pS199 (Fig 6A and 6b), pT205 (Fig 6A and 6c), pT231 (Fig 6A and 6d) and reversed hyperphosphorylation tau at Tau-1 (Fig 6B and 6e). These data indicated the protective role of quercetin in AD pathology.

### Effects of Quercetin on OA-Induced Inhibition of Akt and Activation of GSK-3β

**Fig 7:**
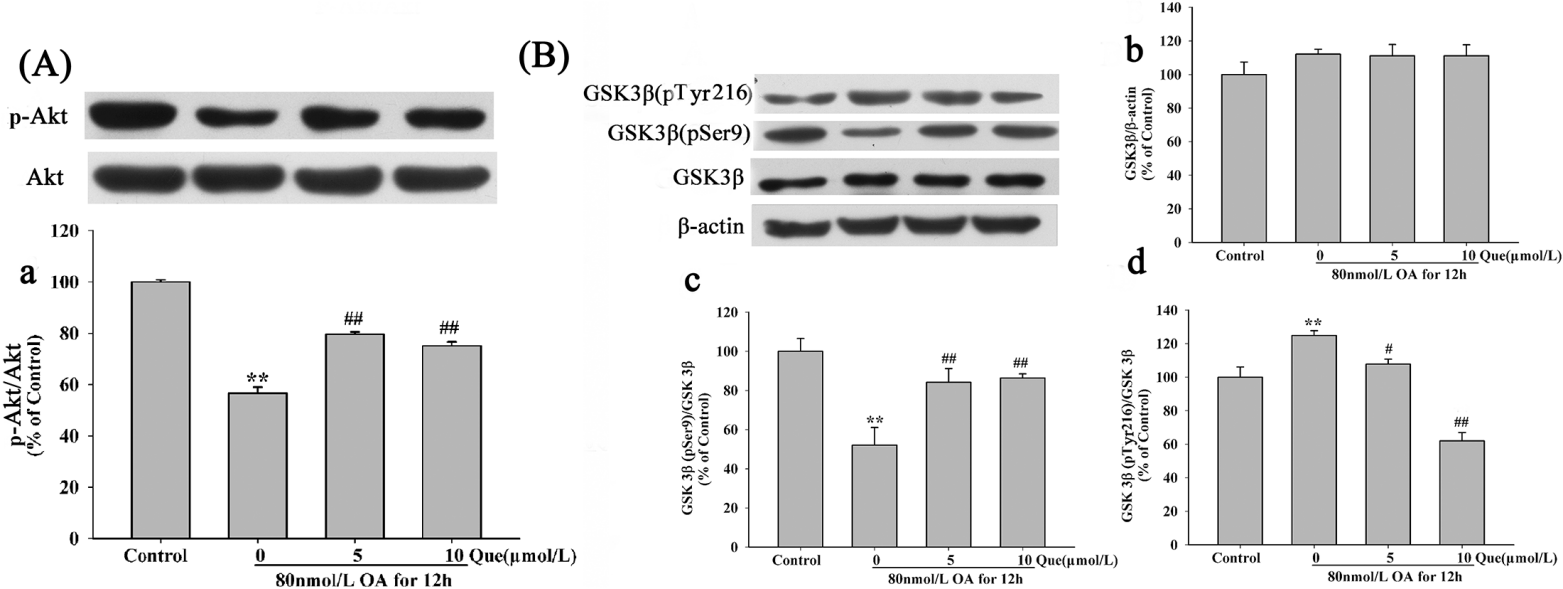
Effects of quercetin and OA induced protein levels of Akt and GSK3β. Cell were treated with 80nmol/L OA for 12h after pre-incubation of quercetin for 12h. Theprotein levels of phosphorylated Akt (**A**) and the phosphorylated GSK3β at pTyr216 (**B**), pSer9 (**B**) and total GSK3β (**B**) were measured by Western blotting. Quantitative analysis for the protein levels was shown in panel **a-d**. (**P < 0.01 *vs*. control, ^#^*P* < 0.05, ^##^*P* < 0.01 *vs*. OA-control).

To investigate the effect of quercetin on inhibition of tau phosphorylation, we measured the level of Akt at Ser473 (the activated form) and GSK-3β at Ser9 (the inactivated form) and Tyr216 (the activated form). It has been shown that pre-treatment with quercetin significantly increased the phosphorylation of Akt at Ser473 (Fig 7A and 7a) and inactivated GSK-3βby the increased phosphorylation of GSK-3β at Ser9 (Fig 7B and 7c) and the decreased phosphorylation of GSK-3β at Tyr216 (Fig 7B and 7d), whereas, the level of total GSK-3β was not affected (Fig 7B and 7b) (The raw data was showed in S2 Fig).

### Effects of Quercetin on Apoptosis-Related Proteins

**Fig 8:**
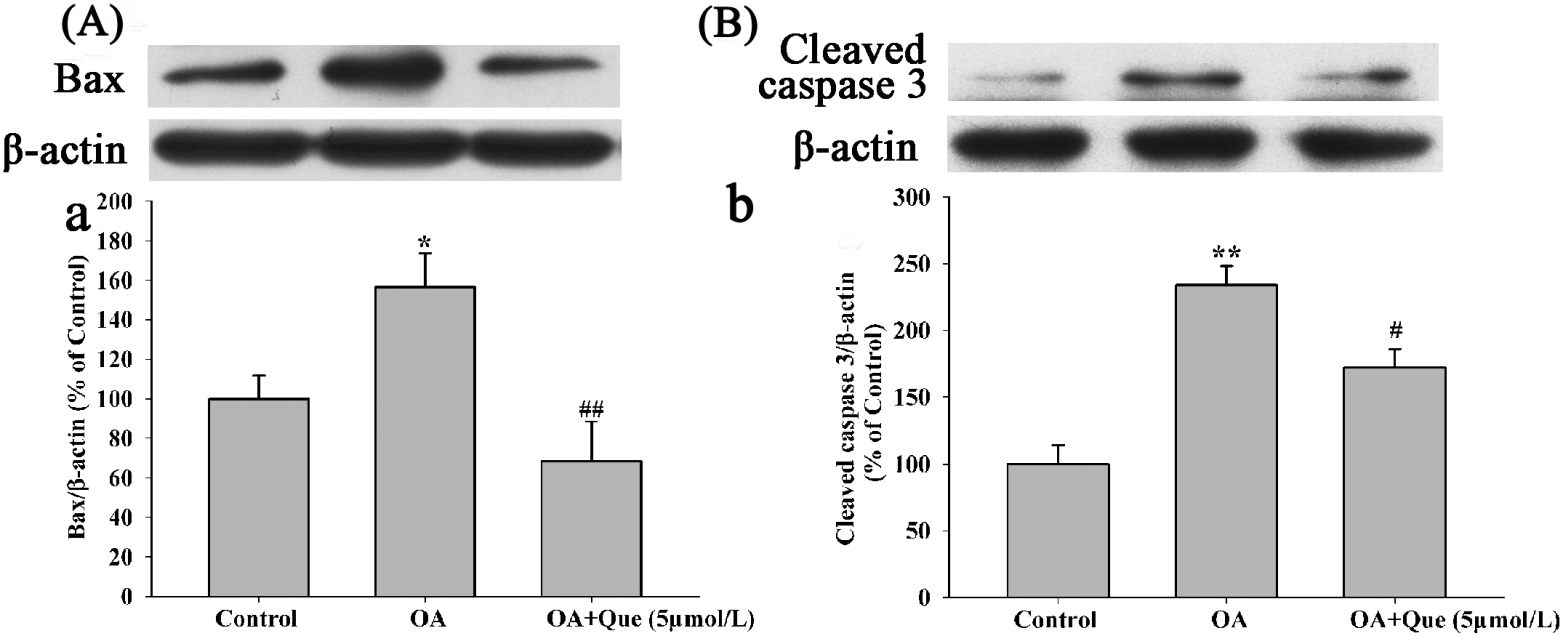
Effects of quercetin on OA induced elevation of cleaved caspase-3 and Bax in HT22 cells. Cleaved caspase-3 **(A**) and Bax (**B**) were measured by western blot. Quantitative analysis of images was shown in panel a and b. Data were analyzed by ANOVA with LSD’s test. (*P < 0.05, **P < 0.01 *vs*. blank control, ^#^*P* < 0.05, ^##^*P* < 0.01 *vs*. OA-control)

The activity of cleaved caspase-3 was significantly elevated in OA (80nmol/L) group compared to control group (Fig 8A and 8a) and quercetin (5μmol/L) reduced the increased activity (Fig 8A and 8a). To further confirm the protective role of quercetin on OA-mediated apoptosis, we investigated the level of Bax. As showed in Fig 8B and 8b, the level of Bax apparently increased in HT22 cells when incubated with 80nmol/L OA and quercetin (5μmol/L) reversed the increased level of Bax (The raw data was showed in S3 Fig).

### Effects of Quercetin on PI3K/Akt/GSK3β Signaling Pathway

**Fig 9:**
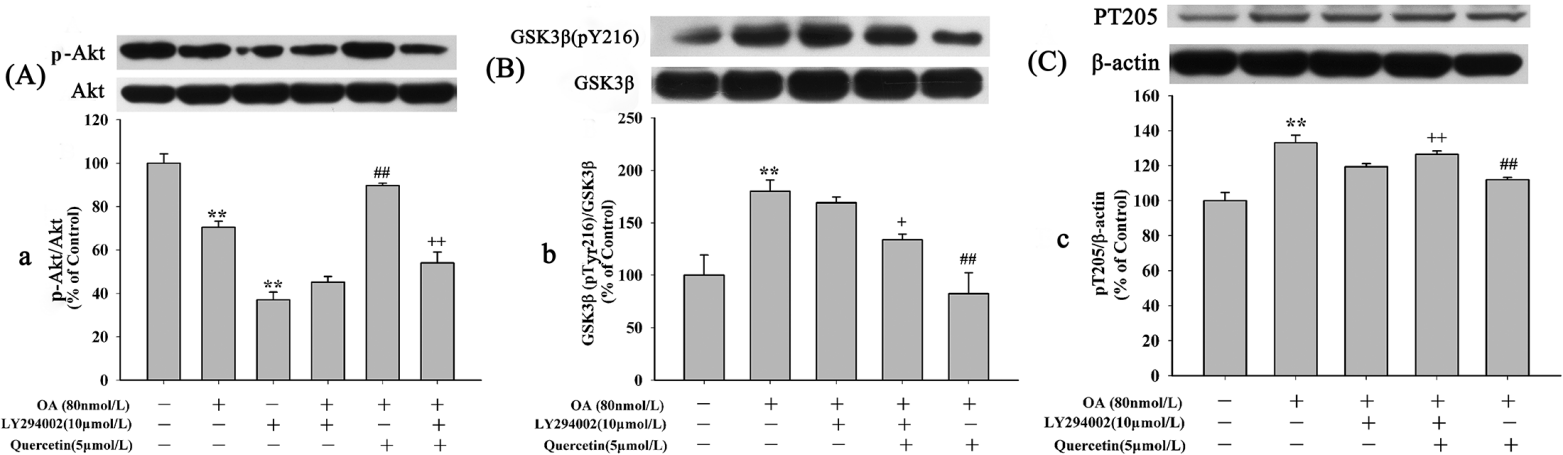
Effects of quercetin and LY294002 on PI3K/Akt/GSK3β Signaling Pathway. HT22 cells were pre-treated with 5μmol/L quercetin or 5μmol/L quercetin plus LY294002 (10μmol/L) for 12 h before exposure to 80nmol/L OA. The protein levels of total Akt, p-Akt (Ser473), total GSK3β, GSK3β (pY216) and PT205 were quantified. We found that pre-treatment of 5μmol/L quercetin decreased the level of GSK3β (pY216) and increased the levels of p-Akt (Ser473) and PT205. However, the effect of quercetin was blocked by 10μmol/L LY294002. Protein levels of phosphorylated GSK3β and Akt were quantified which were normalized by total GSK3β and total Akt respectively. Data were expressed as mean±S.E.M (**P < 0.01 vs. control; ^##^*P* < 0.01 vs. OA-control; ^+^*P* < 0.05, ^++^*P* < 0.01 *vs*. quercetin pre-treatment group).

**Fig 10:**
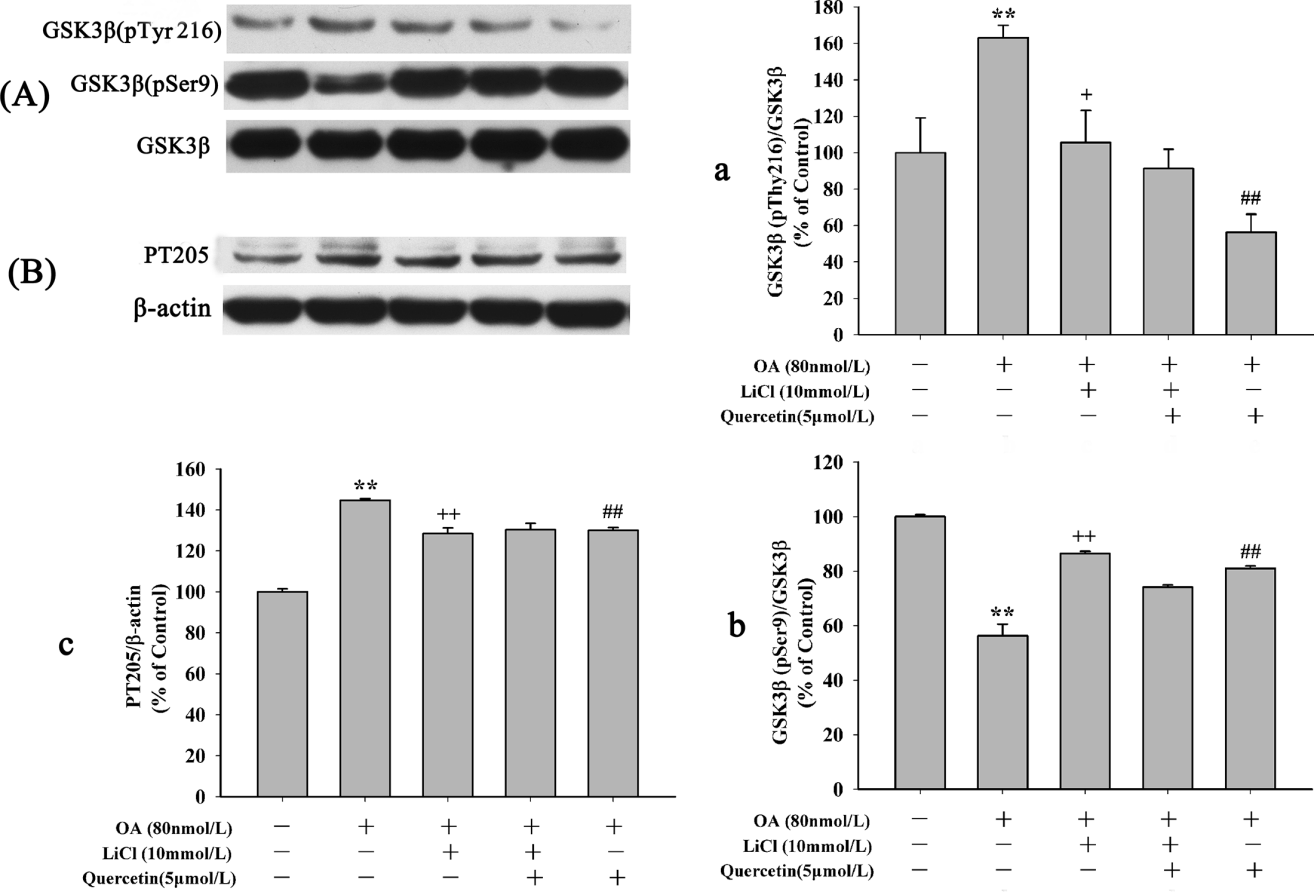
Effects of quercetin and LiCl on PI3K/Akt/GSK3β signaling pathway. HT22 cells were pre-treated with 5μmol/L quercetin or quercetin plus 10mmol/L LiCl for 12h before exposure to 80nmol/L OA. The protein levels of total GSK3β, GSK3β (pSer9), GSK3β (pTyr216) and PT205 were quantified. It revealed that pre-treatment with 5μmol/L quercetin or 10mmol/L LiCl decreased the level of GSK3β (pTyr216) and increased the levels of GSK3β (pSer9) and PT205. Data were expressed as mean±S.E.M (**P < 0.01 *vs*. control; ^##^*P* < 0.01 *vs*. OA-control; ^+^*P* < 0.05, ^++^*P* < 0.01 *vs*. quercetin pre-treatment group).

**Fig 11:**
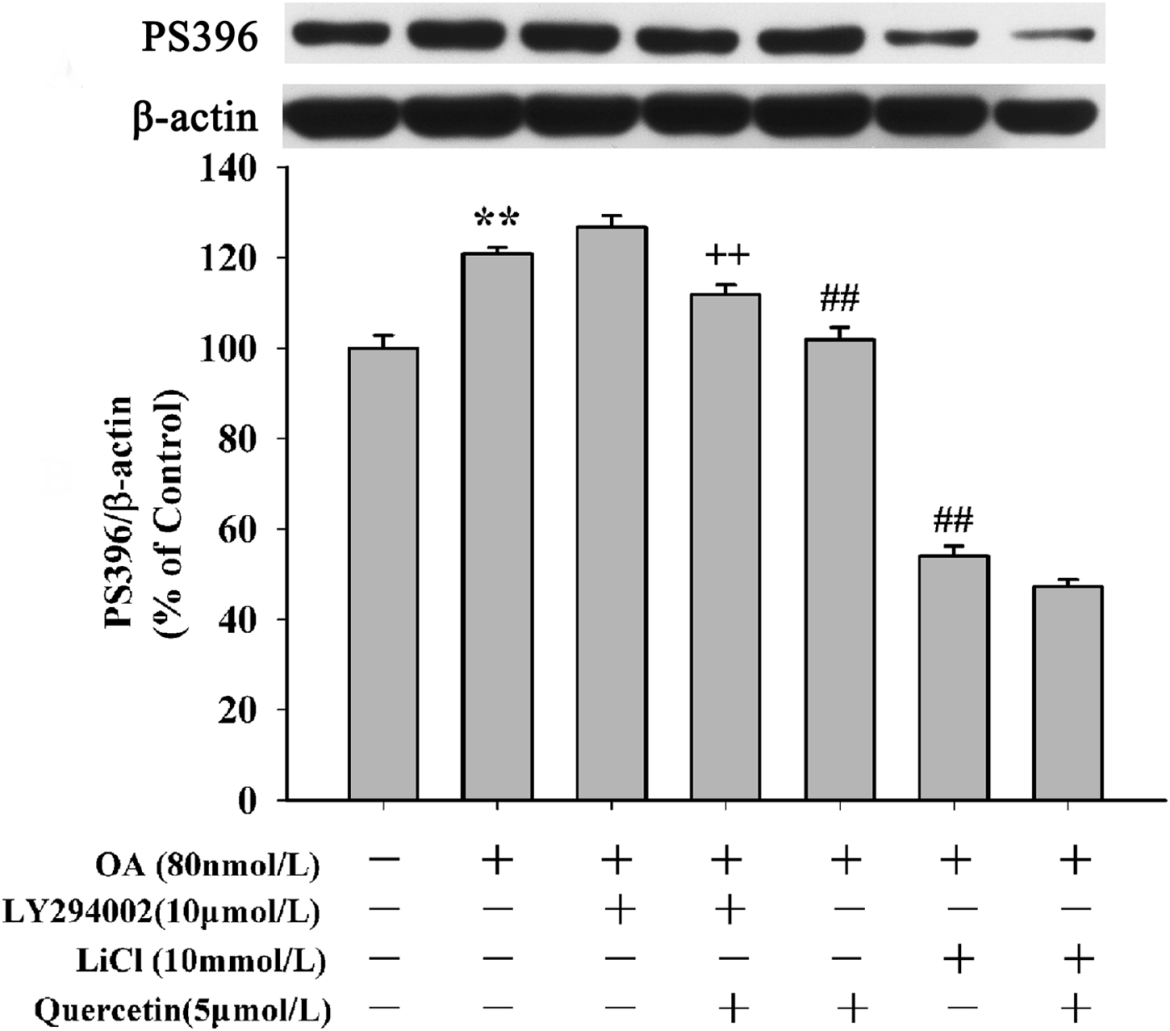
Effects of quercetin, LY294002 and LiCl on PS396. OA (80nmol/L) induced increase of PS396, compared to control group and this effect was reversed by quercetin(5μmol/L) and LiCl (10mmol/L) (n = 3, **P < 0.01 *vs*. control group; ^##^*P* < 0.01 *vs*. OA-control group; ^++^*P* < 0.01 *vs*. Quercetin pre-treatment group).

Akt is the up-stream kinase of GSK3β. Increased GSK3β activity has been implicated in neuronal death. Activation of GSK3β can be mediated by inhibition of PI3K dependent Akt activity [13]. To determine the underlying mechanisms of quercetin on inhibition of apoptosis, the effects of quercetin on PI3K/Akt/GSK3β signaling pathway was studied. As shown in Fig 9A (the second and third lane), cell treated with OA alone or LY294002 (an inhibitor of PI3K/Akt pathway) alone apparently decreased Akt phosphorylation at Ser473, without changing level of total Akt. Pre-treatment with 5μmol/L quercetin reversed the decrease of phosphorylation level of Akt induced by OA(Fig 9A: the fourth lane)and LY294002 blocked quercetin’s effect (Fig 9A: the last lane). We also found OA treatment resulted in a markedly increase of GSK3β (pY216) (Fig 9B) and PT205 (Fig 9C). Pre-treatment with quercetin decreased the elevated expression of GSK3β (pY216) and pT205 induced by OA. Interestingly, compared with quercetin pre-treatment groups, the levels of GSK3β (pY216) and pT205 were decreased in the 10μmol/L LY294002 plus 5μmol/L quercetin pre-treatment group (***P*<0.01**). To further exam the effects of quercetin on PI3K/Akt/GSK3β signaling pathway, cells were pre-treated with LiCl, a GSK3β inhibitor. Our results suggested that OA resulted in a significant increase of GSK3β (pY216)(Fig 10A and 10a) and pT205(Fig 10B and 10c)but the decrease of GSK3β (pSer9) (Fig 10A and 10b). Pre-treatment with 5μmol/L quercetin or 10mmol/L LiCl reversed OA’s effects on the protein levels. The effects of quercetin, LY294002 and LiCl on OA-induced hyperphosphorylation of tau were also be further demonstrated in PS396 (Fig 11). These experiments suggest that quercetin down-regulates the activity of GSK3β via the enhancement of PI3K/Akt.

### Effects of Quercetin on OA-Induced Activation of MAPK Signaling Pathway

**Fig 12:**
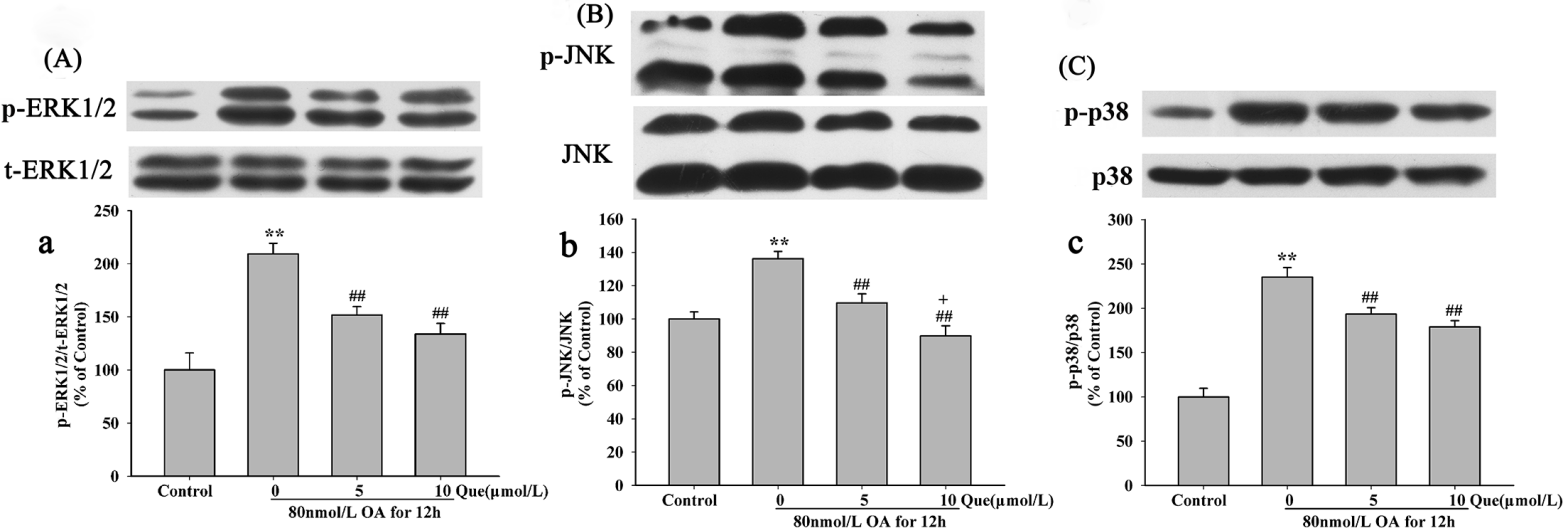
Effects of quercetin on the MAPK pathway in HT22 cells. Protein levels for phospho-ERK1/2/ERK1/2, phospho-JNK/JNK and phospho-p38/p38were shown in panel A-C for OA group and quercetin groups. Quantitative analysis of the bands were shown in panel **a-c**. Data were analyzed by one-way ANOVA with LSD’s post-hoc test and are mean±S.E.M (**P< 0.01 *vs*. control, ^##^*P* < 0.01 *vs*. OA-control).

MAPK signaling pathway mediates a variety of cellular activities in response to extracellular stimuli. ERK1/2, JNK, and p38 are the three components of MAPK which play important roles in cell survival and apoptosis [14, 15]. In the present study, expression levels of both total and phosphorylated proteins were evaluated to determine the involvement of the MAPK signaling pathway in apoptosis. As shown in Fig 12, OA significantly increased the expression of P-ERK1/2(Fig 12A and 12a), P-JNK (Fig 12B and 12b) and p-p38 (Fig 12C and 12c) by 109.31%, 36.21% and 135.29% in HT22 cells, while the levels for total ERK1/2, p38 and JNK were not changed in the absence or presence of OA. Pre-treatment with quercetin significantly inhibited the expression of P-JNK and p-p38 induced by OA. All these results indicate quercetin inhibited OA-induced apoptosis via MAPK signaling pathway in HT22 cells (The raw data was showed in S4 Fig).

### Effects of Quercetin on p65 Subunit of NF-κB

**Fig 13:**
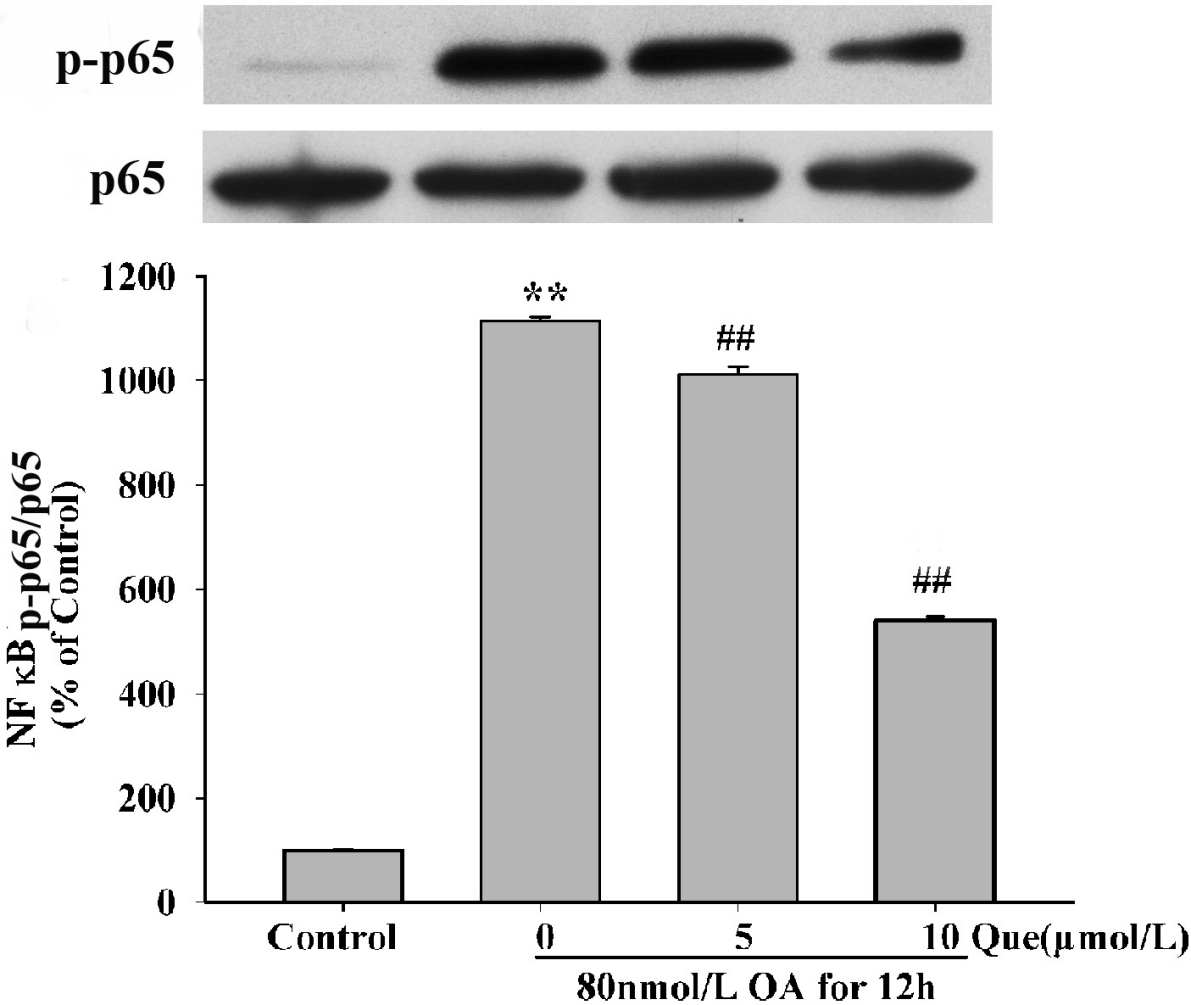
Effects of quercetin on NF-κB activity in OA-treated HT22 cells. OA increased protein level of p-p65 NF-κB and this effect was reversed quercetin (5,10μmol/L) (**A**). Quantitative analysis was shown in the below panel (A). Data are presented as the mean ± S.E.M (**P < 0.01 *vs*. control, ^##^*P* < 0.01 *vs*. OA-control).

NF-κB signaling pathway is an essential pathway involving in multiple cellular responses. We showed that the level of NF-κB p-p65 significantly increased after exposure to 80nmol/L OA for 12h (as shown in Fig 13). This effect was attenuated by prtreatment with quercetin. These data may indicate that quercetin inhibited cell apoptosis via decreasing the transcription activity of NF-κB p65.

## Discussion

The hyperphosphorylation of tau protein is regulated by protein phosphatases and kinases, and PP2A (protein phosphatae 2A) is a key phosphatase [16]. The balance between phosphorylation and de-phosphorylation of tau protein is critical for AD treatment. OA is a specific inhibitor of protein phosphatases 1 and 2A. Hippocampal injection of OA induces the hyperphosphorylation of tau protein through the inhibition of PP2A [4]. In the present study, we determined OA induced phosphorylation of tau, p38-MAPK and GSK3β in a dose-dependent manner which was peaked at 80mmol/L. We see that OA activated GSK3β via phosphorylation of GSK3β at Tyr216, which was supported by previous study that the activity of GSK3β was regulated by phosphorylation of Ser9 and Tyr216 [17]. In addition, the increased phosphorylation of p38-MAPK was also seen after OA induction. OA also resulted in the inhibition of cell viability and increase of LDH release, however, quercetin can reverse these effects and improve cell morphology.

Oxidative stress is due to the imbalance between ROS and anti-oxidative system. Evidence suggests that oxidative stress is a prominent early feature of AD and plays an essential role in the pathogenesis of OA [18, 19]. Some antioxidants, such as GSH and SOD, can scavenge ROS to prevent cell damage. Herein, we investigated the role of quercetin in OA-induced oxidative stress and found that OA significantly inhibited SOD and GSH activities, increased the level of MDA production and the accumulation of ROS. Pre-treatment with quercetin reversed these effects in HT22 cells. These results suggest that quercetin can significantly attenuate OA-induced neurotoxicity by reducing ROS and enhancing the antioxidant system.

Mitochondrial membrane lipids are highly susceptible to ROS, resulting in dysfunction via MMP [20]. We showed a dramatic increase of intracellular ROS but decrease of MMP and pre-treatment with quercetin reversed these effects, indicating the neuroprotective role of quercetin.

Oxidative stress induced tau phosphorylation [21]. An accumulation of 4-HNE, a major production of oxidative stress, hyperphosphorylated tau protein in brain of mouse [22]. In Drosophila, tau-induced neurodegeneration was proved to be mediated by oxidative stress [23]. GSK3β is a downstream enzyme of PI3K/Akt signaling pathway and is considered as the major kinase to phosphorylate tau protein in AD [24]. PI3K activates Akt, which inactivates GSK3β by phosphorylation of Ser9 or dephosphrylation of Tyr216. We showed that quercetin suppressed tau phosphorylation at Ser396, Ser199, Thr205 and Thr231 and increased phosphorylation of tau-1 (dephosphorylated tau at ser195/198/199/202). Moreover, quercetin decreased the activity of GSK3β but increased the activity of Akt (Ser473). These data suggest that the neuroprotective effects of quercetin are associated with anti-hyperphosphorylation of tau protein.

Caspase cascade and Bax family members are key mediators for the apoptotic signaling transduction. Studies showed that hyperphosphorylation of tau and neuronal death were induced by OA andassociated with the induction of Bax in rat brain [25]. The decrease of MMP released an apoptosis inducing factor leading to activation of caspase-3 cascade [7]. Our present study demonstrated that exposure to OA for 12h caused an apparently increase of Bax and caspase-3 activity in HT22 cells. Quercetincompletely reversed the tendency. These data suggest the neuroprotective effects of quercetin was not only because of its antioxidant and anti-apoptotic properties, but also was via the inhibition of apoptosis.

The abnormal tau protein was cleaved by caspase-3 to generate the cleavage of N-terminus, which could activate caspase-8 through FADD and ultimately lead to neuron death [26]. It has been reported that PI3K/Akt/Bad/Bcl signaling pathway involved in Aβ-induced neuronal apoptosis in rat cortex [27]. ROS also plays a critical role in HO-1 expression which was mediated by PI3K/Akt signaling pathway [28]. Therefore, we hypothesized that quercetin inhibited the apoptosis via PI3K/Akt/GSK3β signaling pathway. To confirm this hypothesis, we investigated the effects of LY294002 (PI3K inhibitor) and LiCl (GSK3β inhibitor) on PI3K/Akt/GSK3β signaling pathway. We found that LY294002 blocked the Akt activity induced by quercetin. Furthermore, quercetin also inhibited the activity of GSK3β and LiCl. Our data further support the neuroprotective effects of quercetin was via PI3K/Akt/GSK3β signaling pathway.

It has been reported that chicoric acid induces apoptosis through ROS-mediated PI3K/Akt and MAPK signaling pathways [29]. Our data support that quercetin inhibited, OA induced phosphorylation of ERK1/2, JNK and p38MAPK indicating its neuroprotective role in AD.

NF-κB is a nuclear transcription factor with heterodimers and homodimers structures. It has been reported that ROS induced neuronal apoptosis through the NF-κB and DNA damage pathways in Aβ-infused AD model rats [30]. Oxidative stress activated NF-κB and led to cell death [31] and MAPKs and PI3K/Akt/GSK3β signaling directly or indirectly regulate the activation of NF-κB [32]. In our study, we found that pre-treatment with quercetin inhibited OA induced phosphorylation of NF-κB p65. Therefore, quercetin may paly the anti-apoptotic role via activation of NF-κB p65.

## Conclusions

Our studies investigated the neuroprotective effects and underlying molecular mechanisms of quercetin against OA-induced toxicity in HT22 cells. We found that quercetin markedly attenuated OA-induced hyperphosphorylation of tau protein and oxidative stress and inhibited the neuronal apoptosis via suppression of caspase-3 and Bax activity and activation of NF-κB p65. Furthermore, we demonstrated that the anti-apoptotic role of quercetin was via MAPKs and PI3K/Akt/GSK3β signaling pathways. Our findings support that quercetin may be a potential target for AD treatment.

## Supporting Information

S1 Fig Quercetin reversed OA induced decrease of MMP in HT22 cells. (DOC)

S2 Fig Effects of quercetin and OA induced protein levels of Akt and GSK3β. (DOC)

S3 Fig Effects of quercetin on OA induced elevation of cleaved caspase-3 and Bax in HT22 cells. (DOC)

S4 Fig Effects of quercetin on the MAPK pathway in HT22 cells. (DOC)
