# Supplementary material for: Quercetin Protects against Okadaic Acid-Induced Injury via MAPK and PI3K/Akt/GSK3β Signaling Pathways in HT22 Hippocampal Neurons
Source: PLoS One. 2016 Apr 6;11(4):e0152371. doi: 10.1371/journal.pone.0152371 (PMC4822954; doi:10.1371/journal.pone.0152371)
Supplement: S1 Fig — (DOC) [file pone.0152371.s001.doc]

     Quercetin reversed OA induced decrease of MMP in HT22 cells (200×).  Quercetin was added 12h 
      before OA incubation. The MMP was quantified as the mean intensity of fluorescence. (OA: Okadaic     
      Que: Quercetin)
 
Statistical analysis


Descriptives
	


Rh123	N	Mean	Std. Deviation	Std. Error	95% Confidence Interval for Mean	Minimum	Maximum	
					Lower Bound	Upper Bound			
Control	6	1.000000	0.148624	0.060676	0.844028	1.155972	0.813117	1.171830	
OA80	6	0.419696	0.078707	0.032132	0.337098	0.502294	0.321349	0.520798	
Que 5	6	0.917400	0.168459	0.068773	0.740614	1.094187	0.722868	1.135744	
Que 10	6	1.143248	0.168744	0.068890	0.966161	1.320334	0.951549	1.371010	
Total	24	0.870086	0.309642	0.063205	0.739336	1.000836	0.321349	1.371010	


Multiple Comparisons

Dependent Variable:Rh123	
	(I) Group	(J) Group	Mean Difference (I-J)	Std. Error	Sig.	95% Confidence Interval	
						Lower Bound	Upper Bound	
LSD	Control	OA80	0.580304*	0.084230	0.000001	0.404602	0.756005	
		Que 5	0.082600*	0.084230	0.338484	-0.093102	0.258301	
		Que 10	-0.143248*	0.084230	0.104504	-0.318949	0.032454	
	OA80	Control	-0.580304*	0.084230	0.000001	-0.756005	-0.404602	
		Que 5	-0.497704*	0.084230	0.000009	-0.673405	-0.322003	
		Que 10	-0.723551*	0.084230	0.000000	-0.899253	-0.547850	
	Que 5	Control	-0.082600*	0.084230	0.338484	-0.258301	0.093102	
		OA80	0.497704*	0.084230	0.000009	0.322003	0.673405	
		Que 10	-0.225847*	0.084230	0.014353	-0.401549	-0.050146	
	Que 10	Control	0.143248*	0.084230	0.104504	-0.032454	0.318949	
		OA80	0.723551*	0.084230	0.000000	0.547850	0.899253	
		Que 5	0.225847*	0.084230	0.014353	0.050146	0.401549	
*. The mean difference is significant at the 0.05 level.	
