# Supplementary material for: Quercetin Protects against Okadaic Acid-Induced Injury via MAPK and PI3K/Akt/GSK3β Signaling Pathways in HT22 Hippocampal Neurons
Source: PLoS One. 2016 Apr 6;11(4):e0152371. doi: 10.1371/journal.pone.0152371 (PMC4822954; doi:10.1371/journal.pone.0152371)
Supplement: S2 Fig — (DOC) [file pone.0152371.s002.doc]

                CON: Control; OA: Okadaic  acid; Que: Quercetin£»p-Akt (a,b,c); Akt (d);


Statistical analysis


Descriptives
	


p-Akt/Akt	N	Mean	Std. Deviation	Std. Error	95% Confidence Interval for Mean	Minimum	Maximum	
					Lower Bound	Upper Bound			
Control	3	1.000000	0.012714	0.007341	0.968416	1.031584	0.991760	1.014643	
OA80	3	0.565120	0.041790	0.024128	0.461307	0.668932	0.534856	0.612802	
Que 5	3	0.794970	0.018067	0.010431	0.750090	0.839850	0.778129	0.814053	
Que 10	3	0.749553	0.027123	0.015660	0.682175	0.816932	0.724310	0.778230	
Total	12	0.777411	0.163216	0.047116	0.673708	0.881113	0.534856	1.014643	


Multiple Comparisons

Dependent Variable: p-Akt/Akt	
	(I) Group	(J) Group	Mean Difference (I-J)	Std. Error	Sig.	95% Confidence Interval	
						Lower Bound	Upper Bound	
LSD	Control	OA80	0.434880*	0.022249	0.000000	0.383574	0.486187	
		Que 5	0.205030*	0.022249	0.000016	0.153723	0.256337	
		Que 10	0.250447*	0.022249	0.000003	0.199140	0.301753	
	OA80	Control	-0.434880*	0.022249	0.000000	-0.486187	-0.383574	
		Que 5	-0.229850*	0.022249	0.000007	-0.281157	-0.178544	
		Que 10	-0.184434*	0.022249	0.000034	-0.235740	-0.133127	
	Que 5	Control	-0.205030*	0.022249	0.000016	-0.256337	-0.153723	
		OA80	0.229850*	0.022249	0.000007	0.178544	0.281157	
		Que 10	0.045417*	0.022249	0.075524	-0.005890	0.096723	
	Que 10	Control	-0.250447*	0.022249	0.000003	-0.301753	-0.199140	
		OA80	0.184434*	0.022249	0.000034	0.133127	0.235740	
		Que 5	-0.045417*	0.022249	0.075524	-0.096723	0.005890	
*. The mean difference is significant at the 0.05 level.	


                CON: Control; OA: Okadaic  acid; Que: Quercetin£»GSK3β(pTyr216)(a,b,c,d,e); 
                GSK3β(pSer9) (f,g,h,i); Total GSK3β(j).


Statistical analysis

Descriptives
	


pTyr216/GSK3β	N	Mean	Std. Deviation	Std. Error	95% Confidence Interval for Mean	Minimum	Maximum	
					Lower Bound	Upper Bound			
Control	5	1.000000	0.137138	0.061330	0.829721	1.170279	0.867815	1.220678	
OA80	5	1.248457	0.064379	0.028791	1.168520	1.328394	1.159382	1.321337	
Que 5	5	1.077875	0.067321	0.030107	0.994285	1.161465	0.991260	1.176265	
Que 10	5	0.619890	0.111049	0.049663	0.482004	0.757775	0.506967	0.795477	
Total	20	0.986555	0.253091	0.056593	0.868105	1.105005	0.506967	1.321337	


Multiple Comparisons

Dependent Variable£ºpTyr216/GSK3β	
	(I) Group	(J) Group	Mean Difference (I-J)	Std. Error	Sig.	95% Confidence Interval	
						Lower Bound	Upper Bound	
LSD	Control	OA80	-0.248457*	0.063099	0.001177	-0.382222	-0.114692	
		Que 5	-0.077875*	0.063099	0.234965	-0.211640	0.055890	
		Que 10	0.380110*	0.063099	0.000018	0.246345	0.513875	
	OA80	Control	0.248457*	0.063099	0.001177	0.114692	0.382222	
		Que 5	0.170582*	0.063099	0.015663	0.036818	0.304347	
		Que 10	0.628567*	0.063099	0.000000	0.494803	0.762332	
	Que 5	Control	0.077875*	0.063099	0.234965	-0.055890	0.211640	
		OA80	-0.170582*	0.063099	0.015663	-0.304347	-0.036818	
		Que 10	0.457985*	0.063099	0.000002	0.324220	0.591750	
	Que 10	Control	-0.380110*	0.063099	0.000018	-0.513875	-0.246345	
		OA80	-0.628567*	0.063099	0.000000	-0.762332	-0.494803	
		Que 5	-0.457985*	0.063099	0.000002	-0.591750	-0.324220	
*. The mean difference is significant at the 0.05 level.				
Descriptives	


pSer9/GSK3β	N	Mean	Std. Deviation	Std. Error	95% Confidence Interval for Mean	Minimum	Maximum	
					Lower Bound	Upper Bound			
Control	4	1.000000	0.131732	0.065866	0.790384	1.209616	0.847398	1.167720	
OA80	4	0.520902	0.179995	0.089997	0.234490	0.807314	0.275139	0.703459	
Que 5	4	0.841938	0.140836	0.070418	0.617837	1.066039	0.668579	1.009705	
Que 10	4	0.863834	0.041963	0.020981	0.797062	0.930606	0.832021	0.920606	
Total	16	0.806669	0.217293	0.054323	0.690881	0.922456	0.275139	1.167720	


                  


Multiple Comparisons
Dependent Variable: pSer9/GSK3β	
	(I) Group	(J) Group	Mean Difference (I-J)	Std. Error	Sig.	95% Confidence Interval	
						Lower Bound	Upper Bound	
LSD	Control	OA80	0.479098	0.094437	0.000274	0.273337	0.684859	
		Que 5	0.158062	0.094437	0.120030	-0.047699	0.363823	
		Que 10	0.136166	0.094437	0.174925	-0.069595	0.341927	
	OA80	Control	-0.479098	0.094437	0.000274	-0.684859	-0.273337	
		Que 5	-0.321036	0.094437	0.005275	-0.526797	-0.115275	
		Que 10	-0.342932	0.094437	0.003442	-0.548693	-0.137171	
	Que 5	Control	-0.158062	0.094437	0.120030	-0.363823	0.047699	
		OA80	0.321036	0.094437	0.005275	0.115275	0.526797	
		Que 10	-0.021896	0.094437	0.820557	-0.227657	0.183865	
	Que 10	Control	-0.136166	0.094437	0.174925	-0.341927	0.069595	
		OA80	0.342932	0.094437	0.003442	0.137171	0.548693	
		Que 5	0.021896	0.094437	0.820557	-0.183865	0.227657	
*. The mean difference is significant at the 0.05 level.	


CON: Control; OA: Okadaic  acid; Que: Quercetin£»
Total GSK3β(a,b,c,d); β-actin(e).

                            Statistical analysis


Descriptives
	


Total GSK3β/β-actin	N	Mean	Std. Deviation	Std. Error	95% Confidence Interval for Mean	Minimum	Maximum	
					Lower Bound	Upper Bound			
Control	4	1.000000E0	0.148171	0.074085	0.764228	1.235772	0.879825	1.208437	
OA80	4	1.121572E0	0.057983	0.028991	1.029309	1.213836	1.074909	1.206215	
Que 5	4	1.111967E0	0.133752	0.066876	0.899138	1.324797	0.991844	1.278537	
Que 10	4	1.119904E0	0.131322	0.065661	0.910942	1.328866	0.995538	1.289123	
Total	16	1.088361E0	0.121985	0.030496	1.023360	1.153362	0.879825	1.289123	


Multiple Comparisons

Dependent Variable:Total GSK3β/β-actin	
	(I) Group	(J) Group	Mean Difference (I-J)	Std. Error	Sig.	95% Confidence Interval	
						Lower Bound	Upper Bound	
LSD	Control	OA80	-0.121572	0.086928	0.187259	-0.310972	0.067827	
		Que 5	-0.111967	0.086928	0.222012	-0.301367	0.077432	
		Que 10	-0.119904	0.086928	0.192949	-0.309304	0.069495	
	OA80	Control	0.121572	0.086928	0.187259	-0.067827	0.310972	
		Que 5	0.009605	0.086928	0.913845	-0.179795	0.199004	
		Que 10	0.001668	0.086928	0.985007	-0.187732	0.191067	
	Que 5	Control	0.111967	0.086928	0.222012	-0.077432	0.301367	
		OA80	-0.009605	0.086928	0.913845	-0.199004	0.179795	
		Que 10	-0.007937	0.086928	0.928757	-0.197336	0.181463	
	Que 10	Control	0.119904	0.086928	0.192949	-0.069495	0.309304	
		OA80	-0.001668	0.086928	0.985007	-0.191067	0.187732	
		Que 5	0.007937	0.086928	0.928757	-0.181463	0.197336	
*. The mean difference is significant at the 0.05 level.	
