# Supplementary material for: Quercetin Protects against Okadaic Acid-Induced Injury via MAPK and PI3K/Akt/GSK3β Signaling Pathways in HT22 Hippocampal Neurons
Source: PLoS One. 2016 Apr 6;11(4):e0152371. doi: 10.1371/journal.pone.0152371 (PMC4822954; doi:10.1371/journal.pone.0152371)
Supplement: S3 Fig — (DOC) [file pone.0152371.s003.doc]

       CON: Control; OA: Okadaic  acid; Que: Quercetin£»Cleaved caspase 3:(a,b,c,d); β-acin(e).


Statistical analysis


Descriptives
	


Cleaved Caspase 3/β-actin	N	Mean	Std. Deviation	Std. Error	95% Confidence Interval for Mean	Minimum	Maximum	
					Lower Bound	Upper Bound			
Control	4	0.104991	0.029870	0.014935	0.057461	0.152520	0.065385	0.129316	
OA80	4	0.245622	0.030057	0.015028	0.197795	0.293449	0.204146	0.276096	
Que 5	4	0.180952	0.028749	0.014374	0.135207	0.226698	0.146083	0.215387	
Total	12	0.177188	0.065717	0.018971	0.135434	0.218943	0.065385	0.276096	


Multiple Comparisons

Dependent Variable: Cleaved Caspase 3/β-actin	
	(I) Group	(J) Group	Mean Difference (I-J)	Std. Error	Sig.	95% Confidence Interval	
						Lower Bound	Upper Bound	
LSD	Control	OA80	-0.140631*	0.020905	0.000086	-0.187922	-0.093341	
		Que 5	-0.075961*	0.020905	0.005455	-0.123251	-0.028671	
	OA80	Control	0.140631*	0.020905	0.000086	0.093341	0.187922	
		Que 5	0.064670*	0.020905	0.012856	0.017380	0.111961	
	Que 5	Control	0.075961*	0.020905	0.005455	0.028671	0.123251	
		OA80	-0.064670*	0.020905	0.012856	-0.111961	-0.017380	
*. The mean difference is significant at the 0.05 level.				


           CON: Control; OA: Okadaic  acid; Que: Quercetin£»Bax:(a,b,c,d); β-acin(e).


Descriptives	


Bax/β-actin	N	Mean	Std. Deviation	Std. Error	95% Confidence Interval for Mean	Minimum	Maximum	
					Lower Bound	Upper Bound			
Control	4	1.000000	0.236951	0.118476	0.622958	1.377042	0.787383	1.336892	
OA80	4	1.565771	0.340419	0.170210	1.024087	2.107454	1.192318	2.003245	
Que 5	4	0.682554	0.402265	0.201133	0.042460	1.322648	0.219174	1.181946	
Total	12	1.082775	0.486434	0.140421	0.773709	1.391840	0.219174	2.003245	


Multiple Comparisons

Dependent Variable:Bax/β-actin	
	(I) Group	(J) Group	Mean Difference (I-J)	Std. Error	Sig.	95% Confidence Interval	
						Lower Bound	Upper Bound	
LSD	Control	OA80	-0.565771	0.235884	0.039996	-1.099378	-0.032163	
		Que 5	0.317446	0.235884	0.211296	-0.216162	0.851053	
	OA80	Control	0.565771	0.235884	0.039996	0.032163	1.099378	
		Que 5	0.883217	0.235884	0.004595	0.349609	1.416824	
	Que 5	Control	-0.317446	0.235884	0.211296	-0.851053	0.216162	
		OA80	-0.883217	0.235884	0.004595	-1.416824	-0.349609	
Bonferroni	Control	OA80	-0.565771	0.235884	0.119987	-1.257696	0.126155	
		Que 5	0.317446	0.235884	0.633887	-0.374480	1.009371	
	OA80	Control	0.565771	0.235884	0.119987	-0.126155	1.257696	
		Que 5	0.883217	0.235884	0.013785	0.191291	1.575142	
	Que 5	Control	-0.317446	0.235884	0.633887	-1.009371	0.374480	
		OA80	-0.883217	0.235884	0.013785	-1.575142	-0.191291	
*. The mean difference is significant at the 0.05 level.	
