# Supplementary material for: Quercetin Protects against Okadaic Acid-Induced Injury via MAPK and PI3K/Akt/GSK3β Signaling Pathways in HT22 Hippocampal Neurons
Source: PLoS One. 2016 Apr 6;11(4):e0152371. doi: 10.1371/journal.pone.0152371 (PMC4822954; doi:10.1371/journal.pone.0152371)
Supplement: S4 Fig — (DOC) [file pone.0152371.s004.doc]

          CON: Control; OA: Okadaic  acid; Que: Quercetin£»p-ERK1/2 (a,b,c,d); ERK1/2 (e);


Statistical analysis

Descriptives	


p-ERK1/2/ERK1/2	N	Mean	Std. Deviation	Std. Error	95% Confidence Interval for Mean	Minimum	Maximum	
					Lower Bound	Upper Bound			
Control	4	0.999995	0.322175	0.161087	0.487343	1.512647	0.725080	1.422810	
OA80	4	2.093130	0.197590	0.098795	1.778720	2.407540	1.836680	2.317850	
Que 5	4	1.516482	0.161371	0.080686	1.259705	1.773260	1.335850	1.728280	
Que 10	4	1.338152	0.201470	0.100735	1.017569	1.658736	1.069140	1.556880	
Total	16	1.486940	0.457440	0.114360	1.243187	1.730693	0.725080	2.317850	


Multiple Comparisons

Dependent Variable: p-ERK1/2/ERK1/2	
	(I) Group	(J) Group	Mean Difference (I-J)	Std. Error	Sig.	95% Confidence Interval	
						Lower Bound	Upper Bound	
LSD	Control	OA80	-1.093135*	0.161814	0.000020	-1.445696	-0.740574	
		Que 5	-0.516488*	0.161814	0.007749	-0.869049	-0.163926	
		Que 10	-0.338158*	0.161814	0.058596	-0.690719	0.014404	
	OA80	Control	1.093135*	0.161814	0.000020	0.740574	1.445696	
		Que 5	0.576648*	0.161814	0.003898	0.224086	0.929209	
		Que 10	0.754977*	0.161814	0.000545	0.402416	1.107539	
	Que 5	Control	0.516488*	0.161814	0.007749	0.163926	0.869049	
		OA80	-0.576648*	0.161814	0.003898	-0.929209	-0.224086	
		Que 10	0.178330*	0.161814	0.292044	-0.174231	0.530891	
	Que 10	Control	0.338158*	0.161814	0.058596	-0.014404	0.690719	
		OA80	-0.754977*	0.161814	0.000545	-1.107539	-0.402416	
		Que 5	-0.178330*	0.161814	0.292044	-0.530891	0.174231	
*. The mean difference is significant at the 0.05 level.	


               CON: Control; OA: Okadaic  acid; Que: Quercetin£»p-JNK(a,b,c); JNK (d);
     Statistical analysis
            
Descriptives	
p-JNK/JNK	N	Mean	Std. Deviation	Std. Error	95% Confidence Interval for Mean	Minimum	Maximum	
					Lower Bound	Upper Bound			
Control	3	1.000000	0.073142	0.042229	0.818305	1.181695	0.923895	1.069767	
OA80	3	1.362126	0.075906	0.043824	1.173565	1.550688	1.286376	1.438187	
Que 5	3	1.096985	0.092964	0.053673	0.866050	1.327919	0.991538	1.167113	
Que 10	3	0.898533	0.105757	0.061059	0.635817	1.161249	0.776717	0.966878	
Total	12	1.089411	0.195042	0.056304	0.965487	1.213335	0.776717	1.438187	


Multiple Comparisons

Dependent Variable:p-JNK/JNK	
	(I) Group	(J) Group	Mean Difference (I-J)	Std. Error	Sig.	95% Confidence Interval	
						Lower Bound	Upper Bound	
LSD	Control	OA80	-0.362126*	0.071808	0.000998	-0.527716	-0.196536	
		Que 5	-0.096985*	0.071808	0.213779	-0.262575	0.068605	
		Que 10	0.101467*	0.071808	0.195352	-0.064123	0.267057	
	OA80	Control	0.362126*	0.071808	0.000998	0.196536	0.527716	
		Que 5	0.265141*	0.071808	0.006109	0.099551	0.430731	
		Que 10	0.463593*	0.071808	0.000197	0.298003	0.629183	
	Que 5	Control	0.096985*	0.071808	0.213779	-0.068605	0.262575	
		OA80	-0.265141*	0.071808	0.006109	-0.430731	-0.099551	
		Que 10	0.198452*	0.071808	0.024536	0.032862	0.364041	
	Que 10	Control	-0.101467*	0.071808	0.195352	-0.267057	0.064123	
		OA80	-0.463593*	0.071808	0.000197	-0.629183	-0.298003	
		Que 5	-0.198452*	0.071808	0.024536	-0.364041	-0.032862	
*. The mean difference is significant at the 0.05 level.	


               CON: Control; OA: Okadaic  acid; Que: Quercetin£»p-p38(a,b,c); p38 (d);
 

Statistical analysis


Descriptives	
p-p38/p38	N	Mean	Std. Deviation	Std. Error	95% Confidence Interval for Mean	Minimum	Maximum	
					Lower Bound	Upper Bound			
Control	4	1.000000	0.191531	0.095766	0.695231	1.304769	0.786119	1.204021	
OA80	4	2.352943	0.214417	0.107208	2.011758	2.694128	2.149957	2.607259	
Que 5	4	1.935276	0.144779	0.072389	1.704900	2.165651	1.802219	2.136867	
Que 10	4	1.791660	0.139231	0.069615	1.570113	2.013208	1.599536	1.932715	
Total	16	1.769970	0.529846	0.132461	1.487635	2.052305	0.786119	2.607259	


Multiple Comparisons

Dependent Variable:p-p38/p38	
	(I) Group	(J) Group	Mean Difference (I-J)	Std. Error	Sig.	95% Confidence Interval	
						Lower Bound	Upper Bound	
LSD	Control	OA80	-1.352943*	0.123998	0.000000	-1.623112	-1.082774	
		Que 5	-0.935276*	0.123998	0.000007	-1.205445	-0.665106	
		Que 10	-0.791660*	0.123998	0.000035	-1.061830	-0.521491	
	OA80	Control	1.352943*	0.123998	0.000000	1.082774	1.623112	
		Que 5	0.417667*	0.123998	0.005587	0.147498	0.687837	
		Que 10	0.561283*	0.123998	0.000694	0.291113	0.831452	
	Que 5	Control	0.935276*	0.123998	0.000007	0.665106	1.205445	
		OA80	-0.417667*	0.123998	0.005587	-0.687837	-0.147498	
		Que 10	0.143615*	0.123998	0.269313	-0.126554	0.413784	
	Que 10	Control	0.791660*	0.123998	0.000035	0.521491	1.061830	
		OA80	-0.561283*	0.123998	0.000694	-0.831452	-0.291113	
		Que 5	-0.143615*	0.123998	0.269313	-0.413784	0.126554	
*. The mean difference is significant at the 0.05 level.	
